# Pivotal Clinical Study to Evaluate the Efficacy and Safety of Assistive Artificial Intelligence-Based Software for Cervical Cancer Diagnosis

**DOI:** 10.3390/jcm12124024

**Published:** 2023-06-13

**Authors:** Seongmin Kim, Hyonggin An, Hyun-Woong Cho, Kyung-Jin Min, Jin-Hwa Hong, Sanghoon Lee, Jae-Yun Song, Jae-Kwan Lee, Nak-Woo Lee

**Affiliations:** 1 Gynecologic Cancer Center, CHA Ilsan Medical Center, CHA University College of Medicine, 1205 Jungang-ro, Ilsandong-gu, Goyang-si 10414, Republic of Korea; naiad515@gmail.com; 2 Department of Biostatistics, Korea University College of Medicine, 73 Inchon-ro, Seongbuk-gu, Seoul 02841, Republic of Korea; 3 Department of Obstetrics and Gynecology, Korea University College of Medicine, 73 Inchon-ro, Seongbuk-gu, Seoul 02841, Republic of Korea

**Keywords:** artificial intelligence, cervical cancer screening, colposcopy, deep learning, machine learning

## Abstract

Colposcopy is the gold standard diagnostic tool for identifying cervical lesions. However, the accuracy of colposcopies depends on the proficiency of the colposcopist. Machine learning algorithms using an artificial intelligence (AI) system can quickly process large amounts of data and have been successfully applied in several clinical situations. This study evaluated the feasibility of an AI system as an assistive tool for diagnosing high-grade cervical intraepithelial neoplasia lesions compared to the human interpretation of cervical images. This two-centered, crossover, double-blind, randomized controlled trial included 886 randomly selected images. Four colposcopists (two proficient and two inexperienced) independently evaluated cervical images, once with and the other time without the aid of the Cerviray AI^®^ system (AIDOT, Seoul, Republic of Korea). The AI aid demonstrated improved areas under the curve on the localization receiver-operating characteristic curve compared with the colposcopy impressions of colposcopists (difference 0.12, 95% confidence interval, 0.10–0.14, *p* < 0.001). Sensitivity and specificity also improved when using the AI system (89.18% vs. 71.33%; *p* < 0.001, 96.68% vs. 92.16%; *p* < 0.001, respectively). Additionally, the classification accuracy rate improved with the aid of AI (86.40% vs. 75.45%; *p* < 0.001). Overall, the AI system could be used as an assistive diagnostic tool for both proficient and inexperienced colposcopists in cervical cancer screenings to estimate the impression and location of pathologic lesions. Further utilization of this system could help inexperienced colposcopists confirm where to perform a biopsy to diagnose high-grade lesions.

## 1. Introduction

Cervical cancer is the leading cause of cancer-related mortality and morbidity worldwide [1]. Many cases and deaths occur in low-middle-income countries (LMIC), where prevention programs are limited. Female genital human papillomavirus (HPV) infection is the main cause of cervical intraepithelial neoplasia (CIN) and cancer [2]. CIN is a premalignant cervical cancer lesion categorized as CIN1, CIN2, or CIN3 [3]. Moreover, cervical cancer can be prevented through prophylactic HPV vaccination, screening, and treatment of CIN. Screening for CIN or cervical cancer includes HPV DNA testing, cytology, and visualization using acetic acid [4]. Regular screening for cervical cancer may lower the lifetime risk of the disease [5]. However, screening programs in LMIC are challenging, owing to inaccessibility, lack of funding, lack of public policies, and high costs [6]. Additionally, the dissemination of prophylactic HPV vaccination has been severely limited by a lack of resources and organization [7]. Furthermore, colposcopy is the gold standard diagnostic method for identifying cervical lesions using low-magnification microscopy with acetic acid and Lugol’s solution, with a sensitivity and specificity of 66–96% and 35–98%, respectively, in diagnosing high-grade cervical lesions [8,9]. However, the diagnostic accuracy depends on the skill and proficiency of the colposcopist [10].

Adopting artificial intelligence (AI) in clinical practice may improve healthcare quality and cost-effectiveness [11]. Machine learning algorithms can quickly process large amounts of data and have been successfully applied in several clinical situations [12]. Machine and deep learning models for detecting various diseases, including skin, liver, heart, and Alzheimer were used for the early detection of disease [13]. The different AI techniques (Boltzmann machine, K nearest neighbor, support vector machine, Decision Tree, recurrent neural network, convolutional neural networks (CNN), deep-CNN, generative adversarial networks, and long short-term memory, among others) were applicable in various studies. However, the practical implementation of the models in clinical use is not incorporated. A limited number of previous studies have reported on the feasibility of AI applications in improving the diagnostic quality of high-grade CIN [7,14,15,16]. However, the method of validation of systems is not standardized. Moreover, previous studies compared the impression of the AI system with histologic diagnosis and other conventional screening methods, including cytology and HPV testing. However, how the AI perceives the image and the similarity of the ‘’view’’ between humans and AI have not been evaluated. Therefore, this study aimed to assess the feasibility of an AI system as an assistant tool for diagnosing high-grade CIN lesions compared to human interpretation of cervical images, including both the final impression and the location of the pathologic lesion. This study also compared the effectiveness of AI using professionals and beginners during colposcopies.

## 2. Materials and Methods

### 2.1. Study Design and Terminology

**Figure 1:**
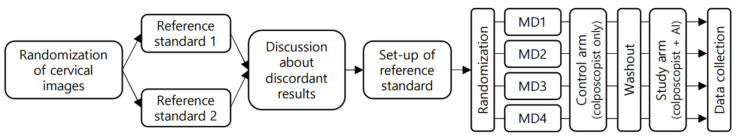
Flowchart of the study. This study is a multicenter, crossover design, double-blind, randomized controlled trial.

This was a multicenter, crossover design, double-blind, randomized controlled trial that evaluated 7457 colposcopy images from two institutions in the Republic of Korea. A complete flowchart of the trial is shown in Figure 1. Patients aged <20 or >50 years were excluded from this study. Additionally, unsatisfactory colposcopic images owing to poor focus or invisible transformational zones were excluded from this study. Patient data and cytological and histopathological results following biopsy were required for inclusion in the study. Other exclusion criteria included a history of surgery on the uterine cervix and total hysterectomy. All 7457 images met the criteria.

Patients were categorized into two or four groups according to the histological results as follows: negative (normal or CIN1) or positive (CIN2/3 or CIN3+) for high-grade lesions. Images were randomly assigned to each group by an independent medical device manager. After randomizing colposcopic images, the ’’reference standard’’ was developed by two professional colposcopists with at least 20 years of clinical experience in colposcopy. Any discordance between the two examiners was discussed and synchronized. Following this setup, the study population was rearranged according to the result of the reference standard.

The same images were interpreted by four colposcopists as follows: two colposcopists (MD1 and MD3) were proficient in colposcopy with 5–10 years of experience, and the others (MD2 and MD4) were relatively inexperienced in colposcopy with less than 5 years of experience. First, the ‘’control’’ interpretation of images was conducted without the assistance of AI software. After 2 weeks of washout, the ‘‘study’’ interpretation was performed with the aid of AI interpretation. Furthermore, data were collected and analyzed after the completion of all interpretations. The primary endpoint of the study was the comparison of the diagnostic value between the control and study interpretations using the localization receiver operating characteristic (LROC) curve. The secondary endpoints of the analysis included the sensitivity for positive results, specificity for negative results, diagnostic accuracy, the concordance rate of interpretation, AI interpretation accuracy, and AI receiver operating characteristic (ROC) curve.

Additionally, liquid-based cytology results were obtained. Histological results were acquired from the pathologic report of the biopsy, which a professional pathologist at both institutions diagnosed. Colposcopic images only included cervical images with acetic acid applied to the cervix; images with Lugol’s solution applied to the cervix were excluded. Our institutional review board approved this study (2021-08-001). The Bethesda and CIN classification systems were used for the cytological and histological evaluations, respectively. The International Federation for Cervical Pathology and Colposcopy Terminology was used to determine the colposcopic impressions.

### 2.2. Preparation of Machine Learning System

Cervical imaging was interpreted using the Cerviray AI^®^ machine learning system (AIDOT, Seoul, Republic of Korea), which was constructed with over 30,000 colposcopy images introduced to the learning algorithm. A multi-category deep learning method was used by integrating a knowledge-based clinical decision support system (CDSS) using clinical colposcopy findings, histopathological results, and a non-knowledge-based CDSS via machine learning. The Cerviray AI^®^ deep learning system comprises three main modules, as described in our previous report [16].

### 2.3. Interpretation of Colposcopic Images

Colposcopic interpretations from the colposcopists and AI system were categorized into “normal,” “CIN1,” “CIN2/3,” or “CIN3.” These findings were also rearranged into the following two groups: negative (normal or CIN1) or positive (CIN2/3 or CIN3+).

### 2.4. Statistical Analysis

The study populations were estimated based on the cutoff value from the hierarchical summary ROC curve for the estimated sensitivity and specificity of control interpretations (0.861 and 0.711, respectively). Additionally, the sensitivity and specificity of the study interpretations were estimated to be 0.930 and 0.890, respectively, according to a report submitted to the Korean Telecommunications Technology Association by AIDOT. Based on this estimation, the proportions of the positive and negative groups were calculated using a mathematical formula (Figure S1). From the formulation results, the ratio of positive to negative groups was decided as 0.800:0.200. Based on a significance level and power of 5% and 80%, respectively, the sample size was calculated using MedCalc version 19.6.415. The calculation recommended 886 images (89 benign, 89 CIN1, 354 CIN2/3, and 354 CIN3+ images) to have sufficient power for evaluation.

The accuracy of the diagnoses was assessed in the validation set using ROC curves created by plotting sensitivity against the false-positive rate and its summary statistic, namely, the area under the curve (AUC). For the LROC curve, which plots the number of true lesion localizations (sensitivity) against that of false-positive localizations per image at various confidence levels or cutoff scores, the images were categorized into 2 × 2 sections [17]. At least two localization matches with the reference standards were required for determining a ‘’positive’’ localization. The Dorfman–Berbaum–Metz method was used to perform an analysis of variance for multi-reader multi-case ROC experiments for the four different colposcopists.

The assumptions of standard normal distributions were verified using Kolmogorov–Smirnov test. Student’s t-test and Mann–Whitney U test were used to analyze parametric and non-parametric variables, respectively. Differences between proportions were compared using Fisher’s exact or Chi-square (χ^2^) test. Pearson’s correlation coefficient was used to compare the correlations between the diagnostic tools. Statistical significance was set at *p* < 0.05. Statistical analysis was performed using SAS 9.4 (SAS Institute, Inc., Cary, NC, USA), R (ver 3.6) ‘’RJafroc packages,’’ R (ver 4.1.3) “meta packages.’’

## 3. Results

### 3.1. Patient and Disease Characteristics

From the original 7457 images, randomization was performed for each image until the number of data reached 886 images with a satisfactory group population. Subsequently, these images were rearranged according to the reference standard (Table 1). The patient characteristics according to the reference standard are presented in Table 2. 

### 3.2. Primary Endpoint

**Figure 2:**
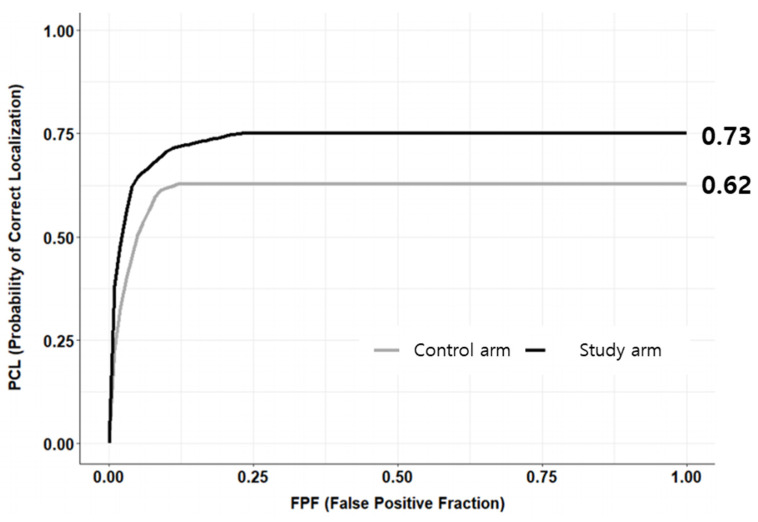
Overall LROC curve from four colposcopists. Study interpretation resulted in better performance than the control diagnosis. (0.73 vs. 0.62, *p* < 0.001, 95% CI [0.10, 0.14]). LROC, localization receiver operating characteristic.

The AUC of the LROC curves is presented in Table 3. MD1 showed no significant difference in the AUC between the control and study groups (*p* = 0.892, 95% confidence interval (CI) [−0.03, 0.04]). MD3 and MD4 demonstrated improvement in the AUC in study interpretation compared to the control diagnosis (*p* < 0.001, 95% CI [0.12, 0.20] and *p* < 0.001, 95% CI [0.34, 0.42], respectively). The overall results using the DBM method showed that the study interpretation resulted in a better performance than the control diagnosis (0.73 vs. 0.62, *p* < 0.001, 95% CI [0.10, 0.14]) (Figure 2).

### 3.3. Secondary Endpoints

The distribution of the colposcopic interpretations of each clinician and the reference standard are presented in Table S1. Table 3 shows the sensitivity and specificity of the control and study diagnoses by each interpreter for high-grade lesions. The overall sensitivity and specificity improved in the study diagnosis compared to the controls (89.18% vs. 71.33%, *p* < 0.001; 96.68% vs. 92.16%, *p* < 0.001, respectively). The diagnostic accuracy of the interpreters also improved on average (86.40% vs. 75.45%, *p* < 0.001) (Table S2). Table S3 shows the diagnostic accuracy of colposcopists according to colposcopic diagnosis. These values also improved for all diagnoses (*p* < 0.05). The diagnostic accuracy of AI interpretation alone for reference standard was 0.93 (95% CI, 0.9–0.95). The AUC of the ROC curve for the AI diagnosis alone was 0.95.

## 4. Discussion

To the best of our knowledge, this is the first study to evaluate the diagnostic value of AI assistance in combination with human interpretation of colposcopic images using the LROC curve. The result of this study implies that the AI assistance not only helped distinguish high-grade lesions from low-grade lesions or normal cervix but also localized the pathologic region. Further utilization of this system could help inexperienced colposcopist confirm where to perform the biopsy to diagnose high-grade lesions. 

Several studies have reported on the feasibility of AI applications for the colposcopic classification of CIN and cervical cancer. The accuracy of the validation dataset was reported as approximately 50% for classifying CIN3, carcinoma in situ, and invasive cancer in 158 patients [18]. Although the study demonstrated the feasibility of AI applications, its diagnostic accuracy was unsatisfactory. Another study reported an accuracy of 72% for the colposcopic images [19]. However, the clinical significance of these results appears limited as only a few images were used to train the machine learning system. Recently, a large-scale study, including 9406 women, demonstrated improved diagnostic accuracy with a deep-learning-based AI system compared with human interpretations or conventional cytology [7]. Furthermore, Cho et al. evaluated AI deep learning models for classifying cervical neoplasms using colposcopic images [20]. The AI demonstrated a diagnostic value comparable to that of human colposcopic impressions. These previous studies were limited because the colposcopic findings were retrospective data derived from multiple colposcopists with varying experiences at various times. We performed a preliminary study that compared colposcopic impressions from two experienced colposcopists with the AI interpretation of CIN [16]. In this study, two proficient gynecologic oncologists separately examined all images. The Cerviray^®^ (AIDOT) system achieved better sensitivity and comparable positive-predictive value in predicting high-grade lesions than the gold standard evaluation method for biopsy based on colposcopy. However, the study population was unbalanced. Additionally, most published studies, including our previous study, used histological results to evaluate the value of diagnostic tools. Therefore, we designed a detailed flowchart to estimate the study population and used a reference standard to compare the diagnostic value of colposcopists and the AI system. This implies that we could evaluate the extent to which the perception of an AI system resembles human visualization.

A colposcopy-assisted biopsy is the primary method used to diagnose precancerous or invasive cervical lesions. However, even physicians who are proficient in colposcopies have difficulties making correct interpretations [21]. The diagnostic accuracy of colposcopies for high-grade cervical lesions varies widely [22]. Therefore, inexperienced colposcopists may miss high-grade lesions. The standardized and less fluctuating characteristics of AI could play a role in this area. AI assistance could result in a nonprofessional gynecologist or general physician making more accurate decisions on whether to perform a punch biopsy or transfer the patient to a specialized center. Additionally, the sensitivity and specificity of this study were better than those of visual inspection with acetic acid (VIA) in a previous meta-analysis [23]. These results suggest that deep-learning-based AI aids may be utilized in clinical settings. This is also supported by a recent study that evaluated deep learning models to automatically classify colposcopic images [20]. AI interpretation might play a role as a diagnostic tool for assessing high-grade cervical lesions in the near future, particularly in LMIC, where proficient colposcopists are insufficient. As previously mentioned, colposcopy evaluation involves a learning curve to achieve proficiency [24]. In contrast, AI systems do not require this learning period; therefore, this approach could be helpful for cervical disease screening programs in LMICs. Furthermore, high laboratory equipment costs are required for cytology and HPV testing, as well as a workforce, including pathologists, resulting in high operating costs. Consequently, AI-aided colposcopic evaluation may be a cost-effective option for cervical cancer screening.

The most significant value of this study is that the AI system showed a benefit not only for diagnosing high-grade cervical lesions but also for the localization of the pathologic region on colposcopic images. To analyze the LROC curve, the observer provided an overall rating as to whether the image was abnormal and marked the most suspicious region in the images. The LROC curve usually shows a lower AUC than the ROC curve due to the inclusion of location information. In this study, the AUC of the LROC significantly improved when colposcopists used information from AI interpretation compared with human interpretation alone (0.73 vs. 0.62). This value could not be compared with those of other studies owing to a lack of previous studies. Interestingly, AI assistance did not always improve the LROC curve in the study armed. Both MD1 and MD3 had already been trained for colposcopic evaluation. AI aid was helpful for MD3 rather than for MD1. The results were also discordant between MD2 and MD4, who were not proficient in colposcopy. Surprisingly, MD2 showed poorer diagnostic performance when using AI impressions. This indicates that individual preference to accept AI interpretations may alter diagnostic accuracy. Therefore, further studies using various colposcopists are warranted to verify the overall benefits of AI assistance in diagnosing high-grade cervical lesions.

This study had several limitations. First, colposcopic evaluation usually provides visual information about the exocervix. Therefore, patients with endocervical lesions are not considered good candidates for accurate evaluation. Inadequate colposcopic findings usually require additional endocervical evaluations, including endocervical cytology or curettage. This can be overcome by HPV co-testing to rule out the possibility of endocervical lesions. Second, this study’s human colposcopic impressions and AI interpretations may not reflect real-time colposcopic diagnoses. Real-time colposcopic diagnosis involves a combination of the visualization of abnormal vascular patterns, the density of acetowhite changes, differences in the degree of acetowhite response, and the degree of light reflection. It also includes color changes after the application of Lugol’s solution. Therefore, the sensitivity and specificity of this study should not be considered a comprehensive colposcopic evaluation. Prospective studies comparing real-time colposcopic impressions with concomitant AI interpretations are required to address this issue. Third, the amount of unknown information regarding HPV infections was relatively high in this study population. However, this study aimed to determine the benefits of AI interpretation for cost-effective exocervical evaluation. The addition of HPV testing could have the benefit of avoiding missed endocervical lesions if the facility or social, medical system is affordable. Therefore, the updated global recommendation for primary HPV testing for cervical cancer should be considered, and further studies on individuals with regular HPV testing should be conducted.

## 5. Conclusions

An AI system could be used as an assistive diagnostic tool for both proficient and inexperienced colposcopists in cervical cancer screening to estimate not only the impression but also the exact location of pathologic lesions. AI interpretation of cervical images could be a beneficial assistive tool to be used in conjunction with human evaluation. Moreover, if additional supportive studies are conducted, it might be utilized as an alternative cost-effective diagnostic tool for evaluating high-grade cervical lesions, particularly in LMICs where proficient colposcopists are not fully available from lack of accessibility or cost. Therefore, further studies using various combinations of screening tools are warranted to determine the significance of AI systems in cervical cancer screening.

## Figures and Tables

**Table 1:** The diagnostic distribution of histologic results and reference standard interpretations. Following the setup of the reference standard, the study population was rearranged accordingly.

		Reference Standard			
		Negative		Positive	
		Normal (*n* = 90)	CIN1 (*n* = 90)	CIN2/3 (*n* = 360)	CIN3+ (*n* = 346)
Histologic diagnosis	Benign (*n* = 89)	86	2	1	0
	CIN1 (*n* = 89)	0	76	12	1
	CIN2/3 (*n* = 354)	0	9	331	14
	CIN3+ (*n* = 354)	4	3	16	331

CIN, cervical intraepithelial neoplasia.

**Table 2:** Patient baseline characteristics according to the distribution of the reference standard.

		Negative Mean (SD)	Positive Mean (SD)	*p*-Value
Age (years)		41.54 (12.98)	41.65 (11.49)	0.917
Parity		1.18 (1.07)	1.22 (1.05)	0.646
HPV	Negative	5 (2.78%)	11 (1.56%)	0.080
	Positive	28 (15.56%)	74 (10.48%)	
	Unknown	147 (81.67%)	621 (87.96%)	

HPV, human papillomavirus; SD, standard deviation.

**Table 3:** Sensitivity and specificity of control and study diagnosis of each interpreter for high-grade lesions. Overall sensitivity and specificity improved in study diagnosis compared to controls.

		MD1	MD2	MD3	MD4	Total
Sensitivity	Control armed	0.79	0.54	0.90	0.96	89.18
		(0.76, 0.82)	(0.51, 0.58)	(0.88, 0.92)	(0.95, 0.98)	(88.12, 90.24)
	Study armed	0.81	0.61	0.75	0.62	71.33
		(0.78, 0.84)	(0.57, 0.65)	(0.72, 0.79)	(0.58, 0.65)	(69.69, 72.97)
	*p*-value	0.424	0.013	<0.001	<0.001	<0.001
Specificity	Control armed	0.96	0.99	0.89	0.78	96.68
		(0.93, 0.99)	(0.97, 1.00)	(0.85, 0.94)	(0.72, 0.84)	(95.42, 97.94)
	Study armed	0.91	0.95	0.92	0.88	92.16
		(0.87, 0.95)	(0.92, 0.98)	(0.88, 0.96)	(0.83, 0.93)	(90.20, 94.11)
	*p*-value	0.052	0.032	0.471	0.012	<0.001

## Data Availability

The data presented in this study are available upon request from the corresponding author.

## Supplementary Materials

The following supporting information can be downloaded at: https://www.mdpi.com/article/10.3390/jcm12124024/s1, Figure S1: The mathematical formula for calculating the proportion of positive and negative groups. Table S1: The distribution of colposcopic interpretation of each clinician and reference standard. Table S2: The diagnostic accuracy of each interpreter. Table S3: Diagnostic accuracy of colposcopists stratified according to the colposcopic diagnosis. The values were also improved in all types of diagnosis.
