# Supplementary material for: Pivotal Clinical Study to Evaluate the Efficacy and Safety of Assistive Artificial Intelligence-Based Software for Cervical Cancer Diagnosis
Source: J Clin Med. 2023 Jun 13;12(12):4024. doi: 10.3390/jcm12124024 (PMC10298986; doi:10.3390/jcm12124024)
Supplement: Supplementary file 1 [file jcm-12-04024-s001.zip › jcm-2363257-supplementary.pdf]

Supplementary materials

Figure S1. The mathematical formula for calculating the proportion of positive and negative groups

$$N = \frac{\left[Z_{\frac{\alpha}{2}}\sqrt{2 \times \bar{P}(1-\bar{P})} + Z_{\beta}\sqrt{P_1(1-P_1) + P_2(1-P_2)}\right]^2}{(P_1 - P_2)^2}$$

P_1 = clinical sensitivity/specificity of control arm

P_2 = clinical sensitivity/specificity of study arm

$\bar{P}$ = average of clinical sensitivity/specificity of both arm

Table S1. The distribution of colposcopic interpretation of each clinician and reference standard

			Reference standard		
			Positive	Negative	Total
			706	180	886
MD1	Study arm	Positive	559	7	566
		Negative	147	173	320
	Control arm	Positive	571	16	587
		Negative	135	164	299
MD2	Study arm	Positive	384	2	386
		Negative	322	178	500
	Control arm	Positive	430	9	439
		Negative	276	171	447
MD3	Study arm	Positive	636	19	655
		Negative	70	161	231
	Control arm	Positive	532	15	547
		Negative	174	165	339
MD4	Study arm	Positive	679	40	719
		Negative	27	140	167
	Control arm	Positive	437	22	459
		Negative	269	158	427

Table S2. The diagnostic accuracy of each interpreter

	MD1	MD2	MD3	MD4	Total

Accuracy	Study arm	0.83 (0.80, 0.85)	0.63 (0.60, 0.67)	0.90 (0.88, 0.92)	0.92 (0.91, 0.94)	86.40 (85.31, 87.49)
	Control arm	0.83 (0.80, 0.85)	0.68 (0.65, 0.71)	0.79 (0.76, 0.81)	0.67 (0.64, 0.70)	75.45 (74.06, 76.85)
	p-value	0.850	0.051	<.001	<.001	<.001
Kappa	Study arm	0.58 (0.53, 0.64)	0.32 (0.28, 0.36)	0.72 (0.67, 0.77)	0.76 (0.71, 0.81)	0.56 (0.54, 0.59)
	Control arm	0.58 (0.52, 0.64)	0.36 (0.31, 0.41)	0.50 (0.45, 0.56)	0.33 (0.28, 0.38)	0.43 (0.40, 0.46)
	p-value	0.879	0.237	<.001	<.001	<.001

Table S3. Diagnostic accuracy of colposcopists stratified according to the colposcopic diagnosis. The values were also improved in all types of diagnosis

		MD1	MD2	MD3	MD4	Total
Normal	Study arm	0.99 (0.97, 1.00)	0.98 (0.95, 1.00)	0.96 (0.91, 1.00)	0.88 (0.81, 0.95)	97.52 (95.94, 99.10)
	Control arm	0.94 (0.90, 0.99)	0.91 (0.85, 0.97)	0.96 (0.91, 1.00)	0.82 (0.74, 0.90)	92.85 (90.23, 95.48)
	p-value	0.097	0.051	>.999	0.297	0.003
CIN1	Study arm	0.52 (0.42, 0.63)	0.79 (0.70, 0.87)	0.70 (0.61, 0.79)	0.48 (0.37, 0.58)	64.32 (59.55, 69.09)
	Control arm	0.48 (0.37, 0.58)	0.67 (0.57, 0.76)	0.38 (0.28, 0.48)	0.47 (0.36, 0.57)	50.04 (45.00, 55.09)
	p-value	0.551	0.066	<.001	0.881	<.001
CIN2/3	Study arm	0.63 (0.58, 0.68)	0.41 (0.36, 0.46)	0.80 (0.76, 0.84)	0.91 (0.88, 0.94)	76.92 (74.96, 78.88)
	Control arm	0.54 (0.48, 0.59)	0.52 (0.47, 0.57)	0.64 (0.59, 0.69)	0.50 (0.45, 0.55)	54.98 (52.43, 57.53)
	p-value	0.010	0.003	<.001	<.001	<.001
CIN3+	Study arm	0.61 (0.56, 0.66)	0.33 (0.28, 0.38)	0.49 (0.44, 0.54)	0.58 (0.53, 0.64)	49.97 (47.41, 52.54)
	Control arm	0.75	0.34	0.32	0.25	41.84

	(0.71, 0.80)	(0.29, 0.38)	(0.27, 0.37)	(0.20, 0.29)	(39.48, 44.20)
p-value	<.001	0.872	<.001	<.001	<.001

CIN, cervical intraepithelial neoplasia
